# Performance of 4 Methods to Assess Health-Related Social Needs

**DOI:** 10.1001/jamanetworkopen.2025.27426

**Published:** 2025-08-18

**Authors:** Joshua R. Vest, Wei Wu, Megan E. Gregory, Suranga N. Kasturi, Eneida A. Mendonca, Jiang Bian, Tanja Magoc, Shaun Grannis, Cassidy McNamee, Christopher A. Harle

**Affiliations:** 1 Department of Health Policy & Management, Indiana University Richard M. Fairbanks School of Public Health, Indianapolis; 2 Center for Biomedical Informatics, Regenstrief Institute, Indianapolis, Indiana; 3 Department of Psychology, Indiana University, Indianapolis; 4 Department of Health Outcomes & Biomedical Informatics, College of Medicine, University of Florida, Gainesville; 5 Division of Biomedical Informatics, Cincinnati Children’s Hospital Medical Center, Cincinnati, Ohio; 6 Department of Biostatistics and Health Data Science, Indiana University School of Medicine, Indianapolis; 7 Quality and Patient Safety, College of Medicine, University of Florida, Gainesville

## Abstract

**Importance:**

Organizations use health-related social needs (HRSN) information to identify patients in need of referrals, to increase clinician awareness, to improve analytics, and for quality reporting.

**Objective:**

To contrast the performance of screening questionnaires, natural language processing (NLP) of clinical notes, rule-based computable phenotypes, and machine learning (ML) classification models in measuring HRSNs.

**Design, Setting, and Participants:**

This cross-sectional study assessed 4 measurement approaches for 5 HRSNs in parallel. Each approach was treated as a screening test. Data included notes from adult patients treated at primary care clinics in 2 health systems in Indianapolis, Indiana, from January 2022 to June 2023. Data were analyzed from December 2024 to February 2025.

**Exposures:**

Reference standard instruments measured food insecurity, housing instability, financial strain, transportation barriers, and history of legal problems. Participants completed the HRSN screening questions in the electronic health record (EHR). NLP algorithms, gradient-boosted decision tree ML classifiers, and refined versions of human-defined rule-based computable phenotypes were applied to participants’ past 12 months EHR data.

**Main Outcomes and Measures:**

Sensitivity, specificity, area under the curve (AUC), and positive predictive values (PPV) described performance of each approach against the reference standard measures. False-negative rates were used to explore fairness.

**Results:**

Data from a total of 1252 adult patients (407 [32.51%] aged 30 to 49 years; 821 [65.58%] female) were assessed, including 94 (7.51%) who identified as Hispanic, 602 (48.08%) as non-Hispanic Black or African American, and 442 (35.30%) as non-Hispanic White. The screening questions method had the strongest overall performance for food insecurity (AUC, 0.94; 95% CI, 0.93-0.95), housing instability (AUC, 0.78; 95% CI, 0.75-0.80), transportation barriers (AUC, 0.77; 95% CI, 0.74-0.79), and legal problems (AUC, 0.81; 95% CI, 0.77-0.85). The screening questions had poor performance for financial strain (AUC, 0.62; 95% CI, 0.60-0.65). The PPV for screening tools ranged from 0.77 to 0.92, indicating utility for individual-level decision-making. NLP and rule-based computable phenotypes had poor performance. ML classification resulted in higher sensitivities than the other methods. False-negative rates indicated differential, unfair performance for all measurement approaches by gender, race and ethnicity, and age groups.

**Conclusions and Relevance:**

In this cross-sectional study of HRSN measurement, no approach performed strongly for every HRSN, and every approach had indication of unfair performance. These findings suggest that practitioners, health care and public health organizations, researchers, and policymakers who rely on a single method to collect HRSN data will likely underestimate patients’ true social burden.

## Key Points

**Question:**

How do different methods of classification perform in measuring health-related social needs (HRSNs)?

**Findings:**

In this cross-sectional study including 1252 adult patients, compared with reference standard measures, neither screening questionnaires, natural language processing of clinical notes, computable-phenotyping, nor machine learning classifiers approaches performed very well for every HRSN. Every approach showed some potential unfairness, measured as false negatives.

**Meaning:**

These findings suggest that reliance on a single method will underestimate patients’ true social burden.

## Teaser

This cross-sectional study compares the performance of screening questionnaires, natural language processing of clinical notes, rule-based computable phenotypes, and machine learning classification models in measuring health-related social needs among US adult patients.

## Introduction

Health-related social needs (HRSNs) include many adverse patient nonclinical, economic, and social characteristics.^1^ Moreover a growing body of evidence indicates that HRSNs affect outcomes such as morbidity, mortality, utilization, disparities, and costs.^2,3,4,5,6^ Increasingly, health care organizations’ operations use HRSN information to identify patients in need of referral to community partners, to increase clinician awareness of patient needs, to improve analytics, and to adhere to Centers for Medicare & Medicaid Services (CMS) reporting requirements.^5,7,8,9,10^

Multiple options exist for measuring HRSNs, and each has advantages and disadvantages. First, the longest-standing source of patients’ social history is the clinical note.^11^ However, extracting HRSNs from text requires natural language processing (NLP).^12^ Second, multidomain screening questionnaires, like the questions included in Epic Systems electronic health record (EHR) software^13^ or CMS’ Accountable Health Communities screening tool,^14^ measure multiple HRSNs in a single tool. Notably, the psychometrics of these questionnaires is unknown or poor.^15,16^ In contrast to recording patient history or using screening tools, computational and analytic options exist. Computable phenotypes, the logical combination of relevant EHR data elements, could be used to identify patients with HRSNs. Rule-based computable phenotypes draw on practitioner expertise, have been successfully developed for specific conditions, and are transparent.^17^ Relatedly, machine learning (ML) approaches for identifying HRSNs via EHR data are promising.^18,19^ ML approaches may be more accurate than humans but less transparent.^17^

This study sought to determine the comparative performance of NLP, a multidomain screening questionnaire, rule-based computable phenotypes, and ML classification models in measuring patients’ HRSNs. A set of independent instruments with known psychometrics served as the reference standard measures of HSRNs, which enabled this novel comparison. Given the potential for biases in measurement and analytics to perpetuate societal disadvantages,^20^ we investigated the fairness, or the equal performance across groups of each approach, as the false-negative rate.^21^ Assessments of each measurement approach’s accuracy and fairness provide immediate guidance to health care organizations, researchers, and policymakers developing and implementing approaches to measuring and using HRSN data.

## Methods

This cross-sectional study was approved by the Indiana University institutional review board. All participants provided written informed consent. This study is reported following the Standards for Reporting of Diagnostic Accuracy (STARD) reporting guideline.^22^

### Study Design

Using a cross-sectional study design, we assessed 4 approaches to measuring HRSN in parallel against a reference standard. Each approach was treated as a screening test. We selected 5 HRSNs relevant to quality reporting,^23^ routinely present in screening questionnaires, and/or commonly assessed in practice^24^: food insecurity,^25^ housing instability,^26^ financial strain,^27^ transportation barriers,^28^ and history of legal (criminal) problems.^29^

### Setting and Participants

Trained research staff recruited a convenience series of adult primary care patients from 2 health systems in Indianapolis, Indiana, from January 2022 to June 2023. Recruitment and data collection occurred in person during office visits. Participants provided authorization to collect and use their protected health information and consented to completing surveys. Data collection was offered in English and Spanish with a $10 gift card incentive.^16,30^

### Data

The survey included 5 separate instruments to measure HRSNs, which served as our reference standard measures (ie, study outcomes). The instruments were previously published and evaluated and were specific to each HRSN.^31,32,33,34,35^ The survey also included the HRSN screening questions included in the Epic EHR.^36^ Participants self-reported race and ethnicity. Survey responses were linked to participants’ past 12 months of EHR data. We abstracted encounter types, procedure codes, orders, diagnoses, payer history, address history, contact information, clinical notes, and demographics. Missing values (eg, no diagnoses or prior encounters) were classified as zero. The choice of past 12 months mirrored the timeframe used in the EHR screening questions. We linked most recent address to the Area Deprivation Index^37^ and the US Census Bureau’s most recent estimates of median household income. All extracted and derived variables are listed in eTable 1 in Supplement 1.

### Presence of HRSNs

The reference standard instruments provided the respective outcome measurement for each HRSN. We created binary indicators for the presence of each HRSN following the instructions provided by the developers of the reference standard instruments.^31,32,33,34,35^

### Multidomain Screening Questionnaires

The Epic EHR screening questionnaire represented a multidomain screening questionnaire (ie, a single tool designed to assess >1 HRSN). The Epic EHR screening questionnaire is a composite of items selected from different existing screening instruments. We followed vendor instructions for creating binary indicators of a positive screening result for each HRSN. As part of the study, the Epic EHR screening questions were measured at the same time as the reference standards (ie, data collection was independent of clinical workflows).

### NLP

From the participants’ linked clinical notes, we identified the presence of each HRSN using NLP. The NLP algorithms were a set of deterministic state machines specific to each HRSN, which used keywords that described each HRSN and logical rules (eg, negation). The algorithms were developed independent of this project using data from multiple health systems, validated through manual review, and tested for generalizability across health systems (eTable 2 in Supplement 1).^38,39^ If any of a participant’s clinical notes in the past 12 months were identified as including the target HRSN, the participant was classified as having the HRSN per NLP.

### Rule-Based Computable Phenotypes

Because computable phenotypes may be developed by humans or using ML classification techniques,^40^ we applied both approaches. We revised and augmented previously published rule-based computable phenotypes.^30^ A multidisciplinary team identified variables indicative of each HRSN or general socioeconomic status and the Boolean-type logic to identify positive instances of each HRSN from the 12 months of prior EHR data. For this study, HRSNs identified by NLP were added to the phenotypes, and we developed a new computable phenotype for legal problems (eTable 3 in Supplement 1).

### Classification Using ML

We built ML classifiers using XGBoost,^41^ an ensemble-based classification algorithm. We selected XGBoost as our representative classification method as it has demonstrated superior performance to other classification algorithms, like random forest, and handles imbalanced data well. We fit separate models for each HRSN using the variables from the EHR data used in the rule-based phenotypes and the linked area-level measures. Race and ethnicity were not used in any modeling. Our modeling approach used 5-fold cross-validation, a grid search for hyperparameter tuning, and balance adjustments for varying HRSN prevalence. As XGBoost results in a continuous predicted probability score (0 to 1), we used Youden *J* to select the optimal cutpoint to identify positive results.^42^ This approach aligns the ML classification results with the other approaches’ binary classification of HRSNs. Models were developed using Python version 3 (Python Software Foundation) with the scikit-learn library.

### Statistical Analysis

We adopted a diagnostic screening test performance evaluation approach.^43^ Each of the measurement approaches resulted in a binary indicator that a particular patient screened positive for (EHR screening questionnaire), had a history of (NLP), met the definition of (rule-based computable phenotype), or was classified as (ML) having a respective HRSN. For simplicity, we refer to each of these approaches as resulting in a positive or negative screening result. By making all approaches binary, we allow for direct comparisons. The series of binary indicators from our reference standard instruments serve as the outcome measures against which screening results were compared.

We calculated sensitivity, specificity, area under the curve (AUC), and F1 scores. To interpret AUC measures, we followed guidelines that values 0.90 or higher indicate a strong test; 0.80 to 0.89, a good test; 0.70 to 0.79, moderate; and 0.70 or less, poor.^44,45^ To describe performance for individual-level decision-making, we calculated positive predictive values (PPV) and positive likelihood ratios.

Failing to identify patients with HRSNs would disadvantage those patients by reducing opportunities for referrals or connections to appropriate services. Therefore, we used the false-negative rates to explore the fairness of each measurement approach.^46^ We focused on fairness-stratified false-negative rates^47^ and AUC (to assess overall performance) by race and ethnicity (eg, Black, non-Hispanic; Hispanic; White, non-Hispanic; and all others, including American Indian or Alaska Native, Asian, and Native Hawaiian or Other Pacific Islander), by gender (eg, female and all others), and age categories. *P* values were 2-sided, and statistical significance was set at *P* ≤ .05. Analyses were conducted in Stata software version 18 (StataCorp) and R software version 4.3.2 (R Project for Statistical Computing). Data were analyzed from December 2024 to February 2025.

## Results

Data from a total of 1252 adult patients (407 [32.51%] aged 30 to 49 years; 821 [65.58%] female) were assessed, including 602 (48.08%) who identified as Black or African American, non-Hispanic; 94 (7.51%) as Hispanic; and 442 (35.30%) as White, non-Hispanic. The sample was reflective of an urban, adult primary care population (Table 1). According to the reference standard instruments, HRSNs were common. More than 4 in 10 participants reported housing instability (533 participants [42.57%]) or food insecurity (540 participants [43.13%]). Approximately one-third of participants faced financial strain (452 participants [36.10%]) as well as transportation barriers (379 participants [30.27%]). More than 1 in 10 participants (161 participants [12.86%]) reported a legal problem, such as a history of incarceration or current parole.

**Table 1.** Characteristics and Presence of Health-Related Social Needs Among Adult Primary Care Patients Measured by Multiple Approaches, Indianapolis, Indiana

Characteristics	Participants, No. (%) (n = 1252)
Gender	
Female	821 (65.58)
Male	431 (34.42)
Age category, y	
18-29	233 (18.61)
30-49	407 (32.51)
50-65	400 (31.95)
66-99	212 (16.93)
Race and ethnicity (self-reported)	
Black, non-Hispanic	602 (48.08)
Hispanic	94 (7.51)
Other, multiple, or unknown^a^	114 (9.11)
White, non-Hispanic	442 (35.30)
Elixhauser comorbidity index, mean (SD)	2.65 (2.60)
Encounter history, mean (SD), No.	
Inpatient admissions	0.15 (0.68)
Emergency department encounters	0.63 (1.43)
Primary care visits	4.98 (4.41)
Presence of health-related social need^b^	
Food insecurity	540 (43.13)
Housing instability	533 (42.57)
Financial strain	452 (36.10)
Transportation barriers	379 (30.27)
Legal problems	161 (12.86)

^a^ Includes American Indian or Alaska Native, Asian, Native Hawaiian or Other Pacific Islander.

^b^ From external criterion surveys.

### Multidomain Screening Questionnaire

The EHR screening questions had the strongest overall performance of any measurement approach for food insecurity, housing instability, transportation barriers, and legal problems (Table 2). For food insecurity, overall performance was very strong, with high sensitivity (0.96; 95% CI, 0.94-0.97), specificity (0.92; 95% CI, 0.90-0.94), AUC (0.94; 95% CI, 0.93-0.95), and F1 score (0.93; 95% CI, 0.91-0.94). Performance was moderate for housing instability (F1 = 0.73; 95% CI, 0.70-0.75; AUC, 0.78; 95% CI, 0.75-0.80) and transportation barriers (F1 = 0.69; 95% CI, 0.66-0.72; AUC, 0.77; 95% CI, 0.74-0.79). The screening questionnaire performed generally well for legal problems (F1 = 0.71; 95% CI, 0.68-0.73; AUC, 0.81; 95% CI, 0.77-0.85). For financial strain, performance was poor (F1 = 0.41; 95% CI, 0.38-0.44; AUC, 0.62; 95% CI, 0.60-0.65). With the exception of food insecurity, the screening questionnaires tended to be more specific than sensitive, thereby tending to underestimate the prevalence of HRSNs.

**Table 2.** Performance of Multidomain Screening Questionnaires, Natural Language Processing, Rule-Based Phenotypes, and Machine Learning Classification Against Reference Standard Measures of Health-Related Social Needs

Measure	Estimate (95% CI)						
	Prevalence, %	Sensitivity	Specificity	F1	AUC	PPV	Positive likelihood ratio
**Screening questionnaire^a^**							
Food insecurity	45.29 (42.50-48.09)	0.96 (0.94-0.97)	0.92 (0.90-0.94)	0.93 (0.91-0.94)	0.94 (0.93-0.95)	0.90 (0.87-0.92)	11.95 (9.31-15.34)
Housing instability	30.43 (27.89-33.06)	0.62 (0.58-0.66)	0.93 (0.91-0.95)	0.73 (0.70-0.75)	0.78 (0.75-0.80)	0.87 (0.83-0.90)	8.72 (6.62-11.49)
Financial strain	11.10 (9.42-12.97)	0.27 (0.23-0.32)	0.98 (0.96-0.99)	0.41 (0.38-0.44)	0.62 (0.60-0.65)	0.88 (0.81-0.93)	12.13 (7.40-19.88)
Transportation barriers	17.89 (15.81-20.13)	0.55 (50.0-60.3)	0.98 (0.97-0.99)	0.69 (0.66-0.72)	0.77 (0.74-0.79)	0.92 (0.88-0.96)	28.15 (17.43-45.47)
Legal problems	10.78 (9.12-12.63)	0.65 (0.58-0.73)	0.97 (0.96-0.98)	0.71 (0.68-0.73)	0.81 (0.77-0.85)	0.77 (0.69-0.84)	22.28 (15.47-32.09)
**Natural language processing^b^**							
Food insecurity	3.67 (2.70-4.87)	0.07 (0.05-0.10)	0.99 (0.98-1.00)	0.14 (0.12-0.16)	0.53 (0.52-0.54)	0.87 (0.74-0.95)	8.79 (3.75-20.58)
Housing instability	2.96 (2.09-4.05)	0.06 (0.04-0.08)	0.99 (0.98-1.00)	0.11 (0.09-0.13)	0.53 (0.52-0.54)	0.86 (0.71-0.95)	8.24 (3.22-21.04)
Financial strain	3.67 (2.70-4.87)	0.04 (0.03-0.07)	0.97 (0.95-0.98)	0.08 (0.07-0.10)	0.51 (0.49-0.52)	0.44 (0.30-0.60)	1.38 (0.77-2.45)
Transportation barriers	4.31 (3.26-5.59)	0.11 (0.08-0.15)	0.99 (0.98-0.99)	0.20 (0.18-0.22)	0.55 (0.53-0.57)	0.80 (0.67-0.89)	9.00 (4.70-17.27)
Legal problems	3.43 (2.50-4.60)	0.13 (0.08-0.19)	0.98 (0.97-0.99)	0.21 (0.19-0.23)	0.56 (0.53-0.58)	0.50 (0.34-0.66)	6.71 (3.75-12.00)
**Rule-based phenotype^c^**							
Food insecurity	9.35 (7.79-11.09)	0.16 (0.13-0.19)	0.96 (0.94-0.97)	0.26 (0.24-0.29)	0.56 (0.54-0.58)	0.74 (0.65-0.81)	3.66 (2.46-5.43)
Housing instability	5.91 (4.67-7.36)	0.09 (0.07-0.12)	0.97 (0.95-0.98)	0.17 (0.15-0.19)	0.53 (0.52-0.54)	0.69 (0.57-0.79)	2.89 (1.79-4.67)
Financial strain	54.39 (51.59-57.18)	0.69 (0.65-0.73)	0.55 (0.51-0.58)	0.56 (0.53-0.59)	0.62 (0.59-0.65)	0.47 (0.43-0.51)	1.53 (1.39-1.69)
Transportation barriers	5.35 (4.17-6.75)	0.15 (0.11-0.19)	0.99 (0.98-0.99)	0.25 (0.23-0.27)	0.57 (0.55-0.58)	0.82 (0.71-0.90)	10.56 (5.72-19.48)
Legal problems	4.39 (3.33-5.68)	0.15 (0.10-0.21)	0.97 (0.96-0.98)	0.22 (0.20-0.24)	0.56 (0.53-0.59)	0.45 (0.32-0.60)	5.55 (3.32-9.29)
**Machine learning classification**							
Food insecurity	52.80 (49.99-55.59)	0.76 (0.72-0.79)	0.65 (0.61-0.68)	0.68 (0.65-0.70)	0.70 (0.68-0.73)	0.62 (0.58-0.66)	2.14 (1.92-2.39)
Housing instability	47.12 (44.33-49.93)	0.62 (0.58-0.66)	0.65 (0.62-0.69)	0.60 (0.57-0.63)	0.64 (0.61-0.66)	0.57 (0.53-0.61)	1.79 (1.58-2.01)
Financial strain	58.79 (56.00-61.53)	0.79 (0.75-0.83)	0.54 (0.51-0.58)	0.62 (0.59-0.65)	0.67 (0.64-0.69)	0.50 (0.47-0.54)	1.74 (1.59-1.90)
Transportation barriers	44.73 (41.95-47.53)	0.73 (0.68-0.78)	0.68 (0.64-0.71)	0.59 (0.56-0.62)	0.70 (0.68-0.73)	0.50 (0.45-0.54)	2.25 (2.01-2.53)
Legal problems	30.75 (28.20-33.39)	0.68 (0.61-0.75)	0.76 (0.73-0.78)	0.41 (0.38-0.44)	0.72 (0.68-0.76)	0.29 (0.25-0.34)	2.80 (2.41-3.24)

Abbreviations: AUC, area under the curve; PPV, positive predictive value.

^a^ From Epic electronic health record screening questions.

^b^ From existing natural language processing algorithm applied to past 12 months of clinical notes.

^c^ Definitions are provided in eTable 3 in Supplement 1.

For individual-level decision-making, results indicated the screening questions were useful. PPVs for food insecurity (0.90; 95% CI, 0.87-0.92), housing instability (0.87; 95% CI, 0.83-0.90), financial strain (0.88; 95% CI, 0.81-0.93), and transportation barriers (0.92; 95% CI, 0.88-0.96) indicate approximately 9 of 10 participants screening positive were truly positive for the respective HRSN. For legal problems, approximately 8 of 10 participants (PPV, 0.77; 95% CI, 0.69-0.84) screening positive were true positives. The positive likelihood ratios provided further support for the utility for individual decision-making (Table 2).

The screening questionnaires exhibited some differential performance (eTable 4 and eFigures 1-3 in Supplement 1). For all HRSNs except legal problems, the false-negative rate was higher for older (age >66 years) participants. For legal problems, false-negative rates were highest among participants younger than 30 years. Additionally, the AUCs for food insecurity were different by race and ethnicity, with false-negative rates highest for Hispanic participants. AUCs also differed by gender for housing instability and transportation barriers.

### NLP

NLP performed poorly for all 5 HRSNs, with F1 scores ranging from 0.08 to 0.21 and AUCs from 0.51 to 0.56 (Table 2). NLP exhibited higher specificity than sensitivity for all HRSNs. The prevalence of each HRSN per NLP was also very low (<5%).

For individual-level decision-making, extracting food insecurity (PPV, 0.87; 95% CI, 0.74-0.95), housing instability (PPV, 0.86; 95% CI, 0.71-0.95), and transportation barriers (PPV, 0.80; 95% CI, 0.67-0.89) could be useful. Participants with presence of these HRSNs in their clinical notes were likely to be truly positive. NLP could not be used for individual decision-making for financial strain (PPV, 0.44; 95% CI, 0.30-0.60) or legal problems (PPV, 0.50; 95% CI, 0.34-0.66).

AUC values for financial strain, transportation barriers, and legal problems differed by age (eTable 4 and eFigures 1-3 in Supplement 1). AUC values also differed by race and ethnicity for housing instability and legal problems.

### Rule-Based Computable Phenotype

The rule-based computable phenotype method performed no better than coin-flips for food insecurity, housing instability, transportation barriers, and legal problems (Table 2). For each of these HRSNs, specificity was high (≥0.96), sensitivity was low (≤0.16), and prevalence was lower than reported against the reference standard measures. Of all the HRSNs, financial strain had the best performance (F1 = 0.56; 95% CI, 0.53-0.59; AUC, 0.62; 95% CI, 0.59-0.65) with higher sensitivity (0.69; 95% CI, 0.65-0.73).

The food insecurity (PPV, 0.74; 95% CI, 0.65-0.81) and transportation barriers (PPV, 0.82; 95% CI, 0.71-0.90) rule-based phenotypes could potentially be used at for individual-level decision-making. For the remaining HRSNs, the rule-based phenotypes would be very poor at identifying true-positive cases.

False-negative rates varied by age for each HRSN, except for food insecurity (eTable 4 and eFigures 1-3 in Supplement 1). For legal problems and food insecurity, Hispanic participants had higher false negative rates. Housing instability and legal problems AUC values also varied by race and ethnicity.

### ML Classification

ML classification performance was moderate for food insecurity (F1 = 0.68; 95% CI, 0.65-0.70; AUC, 0.70; 95% CI, 0.68-0.73), transportation barriers (F1 = 0.59; 95% CI, 0.56-0.62; AUC, 0.70; 95% CI, 0.68-0.73), and legal problems (F1 = 0.41; 95% CI, 0.38-0.44; AUC, 0.72; 95% CI, 0.68-0.76) (Table 2). While ML classification for financial strain was poor (F1 = 0.62; 95% CI, 0.59-0.65; AUC, 0.67; 95% CI, 0.64-0.69), it outperformed all other methods. The ML classification also resulted in higher sensitivities (0.62 to 0.79) than the other methods. As a result, the prevalence estimates from the ML classification models tended to be consistent or even higher than the reference standard measures.

The ML classification models did not perform well enough to be used in individual-level decision-making. PPVs (ranging from 0.29 to 0.62) were equivalent to coin-flips or worse. The ML classification models demonstrated differential performance by age category for all HRSNs, with false-negative rates highest among older (age >66 years) participants (eTable 4 and eFigures 1-3 in Supplement 1). Differential performance, either by AUC value or false-negative rates, was indicated by race and ethnicity for all HRSN except for transportation barriers. False-negative rates varied by gender for food insecurity and legal problems.

## Discussion

This cross-sectional study of 4 methods for identifying HRSNs from the EHR found that overall, screening questionnaires performed best. However, compared with reference standard measures, none of the measurement approaches performed well for every HRSN. Furthermore, every measurement approach showed some potential unfairness. For researchers and policymakers, the variable performance could have cascading implications for intervention development, quality measurement interpretation, or public health policy formation. For health care delivery organizations and clinicians, these HRSN measurement approaches will have differential usefulness in supporting population health programs or individual patient care.

Individuals screening positive for HRSNs via questionnaire are likely to be true positives, suggesting clinicians and organizations can confidently make patient referral decisions based on questionnaires. This is notable because many organizations already use multidomain screening questions to drive referrals.^48^ While the overall performance metrics for food insecurity screening questions were strong and the legal problems screening questionnaires were good, every other HRSN performed below what is generally considered useful.^44^ Screening questionnaires missed approximately 40% or more of true-positive housing instability, financial strain, and transportation barriers. As a result, organizations, policymakers, and researchers may not be able to rely on questionnaires to understand the burden and distribution of HRSNs at population levels. The poor population-level accuracy is disappointing because many have viewed screening questionnaires as a solution to the underutilization of *International Statistical Classification of Diseases and Related Health Problems, Tenth Revision* (*ICD-10*) *Z* codes.^49^ The large number of incorrectly classified individuals is consequential, given that HRSNs could be used as adjustments in severity coding, in hospital reimbursements, or for public quality reporting.^7,50,51^ Housing instability and transportation barriers are included in CMS’s quality measures, but these findings suggest screening questionnaires may be insufficiently accurate for these HRSNs.

Likewise, using NLP to classify clinical documents has strengths and weaknesses. The presence of food insecurity, housing instability, and transportation barriers in clinical notes was frequently indicative of a true HRSN. This value for individual-level decision-making is consistent with other studies of NLP for HRSNs that have demonstrated strong PPVs.^52^ Problematically, like other studies, HRSNs did not appear frequently in the clinical notes in this study.^52,53,54^ To appear in notes, HRSNs must be volunteered by patients and clinicians must opt to document the need. Thus, because of their low prevalence, HRSN extracted from NLP may supplement other forms of data collection, but likely cannot be a standalone approach.

ML classification models did not have strong performance but tended to be more sensitive than other approaches. As a more sensitive method, ML classification could pair well with the high-specificity screening questionnaires in a 2-stage screening strategy. Because health care organizations need to screen large numbers of patients, such a 2-stage screening approach could be effective and efficient.^5^ Like ML classification, rule-based phenotype performance was poor, and inclusion of new features did not improve on prior performance.^30^ This is disappointing because EHRs contain abundant structured data that may be useful, and phenotype approaches to screening could be efficient and scalable. While the performance of our ML classification models was moderate at best, they were consistent with or better than what is reported in the literature^18,19^; however, direct comparison of performance with other studies is challenging because others have not used reference standard HSRN measures. The poorer performance of these 2 approaches may be due to the extended timeframe covered by the underlying data or data quality issues.

Finally, our findings amplify concerns about unfairness for individuals most in need.^55^ Measuring fairness as false-negative rates, we found indications of unfair performance across the HRSNs assessed. The most consistent differences were by age group and then by race and ethnicity. For ML classification, computable phenotypes, and even NLP, differences could be driven by different patterns of health care utilization by age, race, and ethnicity. Differential access to care is a common source of bias in data that are generated during the normal course of health care delivery, and computational methods for addressing bias in ML exist.^56^ The differences in performance in the screening questionnaires are difficult to explain and potentially more problematic, given their use in CMS quality metrics.^7^ Our study is not designed to identify the drivers of this differential performance but highlights this important variation for further investigation. The findings suggest organizations using this information as part of population health management strategies should implement quality monitoring processes to ensure consistent referral rates across patient populations.

### Limitations

This study has some limitations. This study was limited to a convenience sample of individuals who visited primary care and may not generalize to other delivery settings. Data were collected as a part of a research study; participants may respond differently if screened by clinical care practitioners. Our findings do not generalize to alternative measurement strategies, such as categorical instead of binary outcomes, or using large language models for classification. Additionally, our ML classifier and human-defined rule-based computable phenotypes may have limited generalizability. Other ML classifiers may have different performance. Performance could be explained by other issues: the measurement approaches and the reference standard instruments may measure different HRSNs constructs, the reference standard measures also have measurement error, or collecting all survey data simultaneously may have affected responses.

## Conclusions

This cross-sectional study found that no measurement approach performed strongly for every HRSN, and every measurement approach had an indication of unfair performance. Practitioners, including health care and public health organizations, researchers, and policymakers, who rely on a single method to collect HRSN data will likely underestimate patients’ true social needs burdens.

## Notes

Supplement 1. **eFigure 1.** False negative rates by gender for each health-related social need and measurement approach **eFigure 2.** False negative rates by race & ethnicity for each health-related social need and measurement approach **eFigure 3.** False negative rates by age category for each health-related social need and measurement approach **eTable 1.** List of reference standard outcomes, screening variables, and features used in ML classification and rule-based computable phenotype construction **eTable 2.** Prior performance of natural language processing algorithms **eTable 3.** Rule-based health-related social needs computable phenotype definitions **eTable 4.** Differential performance of measurement approaches for health-related social needs by demographic groups

Supplement 2.
Data Sharing Statement
