# Supplementary material for: Performance of 4 Methods to Assess Health-Related Social Needs
Source: JAMA Netw Open. 2025 Aug 18;8(8):e2527426. doi: 10.1001/jamanetworkopen.2025.27426 (PMC12362220; doi:10.1001/jamanetworkopen.2025.27426)
Supplement: Supplement 1. — eFigure 1. False negative rates by gender for each health-related social need and measurement approach eFigure 2. False negative rates by race & ethnicity for each health-related social need and measurement approach eFigure 3. False negative rates by age category for each health-related social need and measurement approach eTable 1. List of reference standard outcomes, screening variables, and features used in ML classification and rule-based computable phenotype construction eTable 2. Prior performance of natural language processing algorithms eTable 3. Rule-based health-related social needs computable phenotype definitions eTable 4. Differential performance of measurement approaches for health-related social needs by demographic groups [file jamanetwopen-e2527426-s001.pdf]

Supplemental Online Content

Vest JR, Wu W, Gregory ME, et al. Performance of 4 methods to assess health-related social needs. *JAMA Netw Open*. 2025;8(8):e2527426. doi:10.1001/jamanetworkopen.2025.27426

eFigure 1. False negative rates by gender for each health-related social need and measurement approach

eFigure 2. False negative rates by race & ethnicity for each health-related social need and measurement approach

eFigure 3. False negative rates by age category for each health-related social need and measurement approach

eTable 1. List of reference standard outcomes, screening variables, and features used in ML classification and rule-based computable phenotype construction

eTable 2. Prior performance of natural language processing algorithms

eTable 3. Rule-based health-related social needs computable phenotype definitions

eTable 4. Differential performance of measurement approaches for health-related social needs by demographic groups

This supplemental material has been provided by the authors to give readers additional information about their work.

© 2025 Vest JR et al. *JAMA Network Open*.

Efigure 1. False negative rates by gender for each health-related social need and measurement approach.

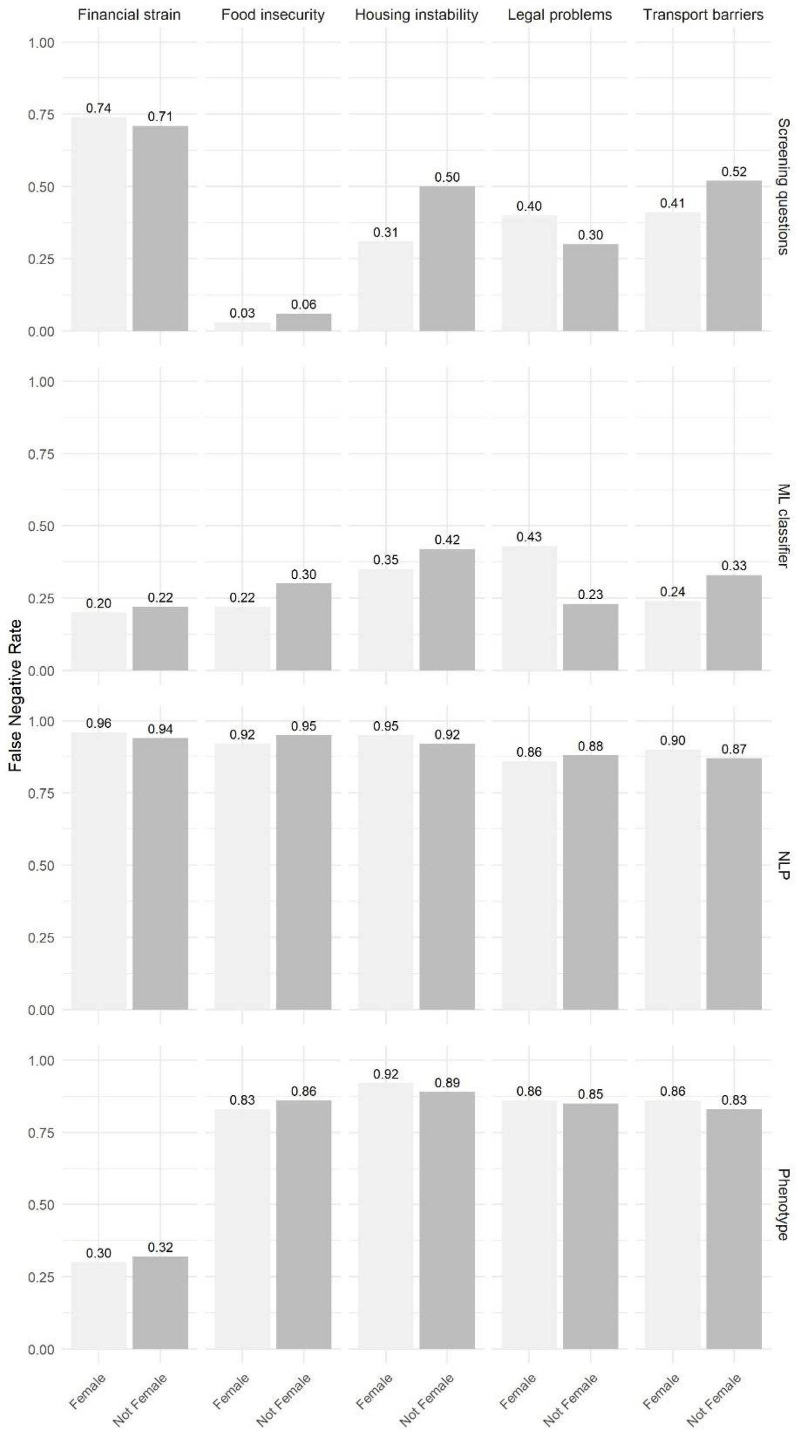

© 2025 Vest JR et al. *JAMA Network Open*.

Efigure 2. False negative rates by race & ethnicity for each health-related social need and measurement approach.

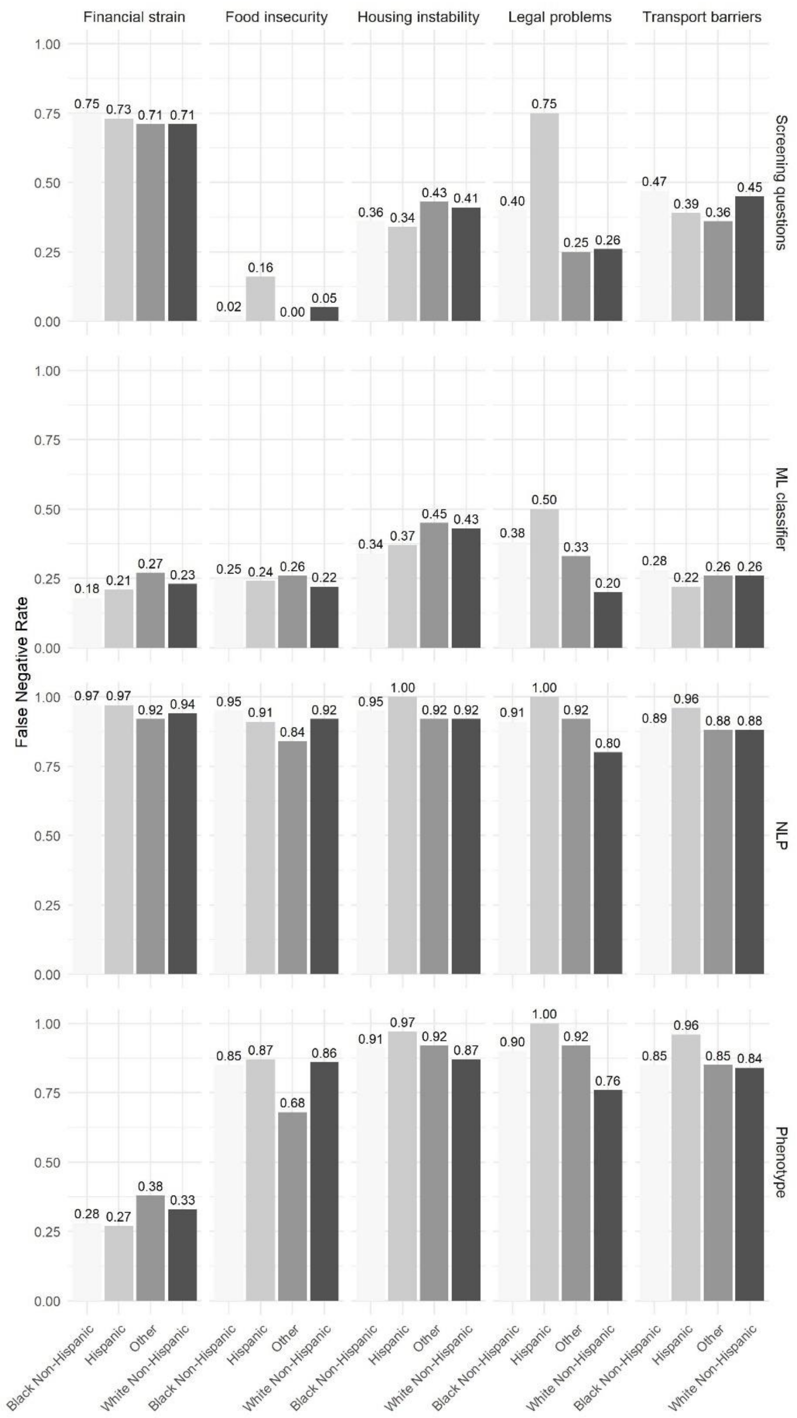

© 2025 Vest JR et al. *JAMA Network Open*.

efigure 3. False negative rates by age category for each health-related social need and measurement approach.

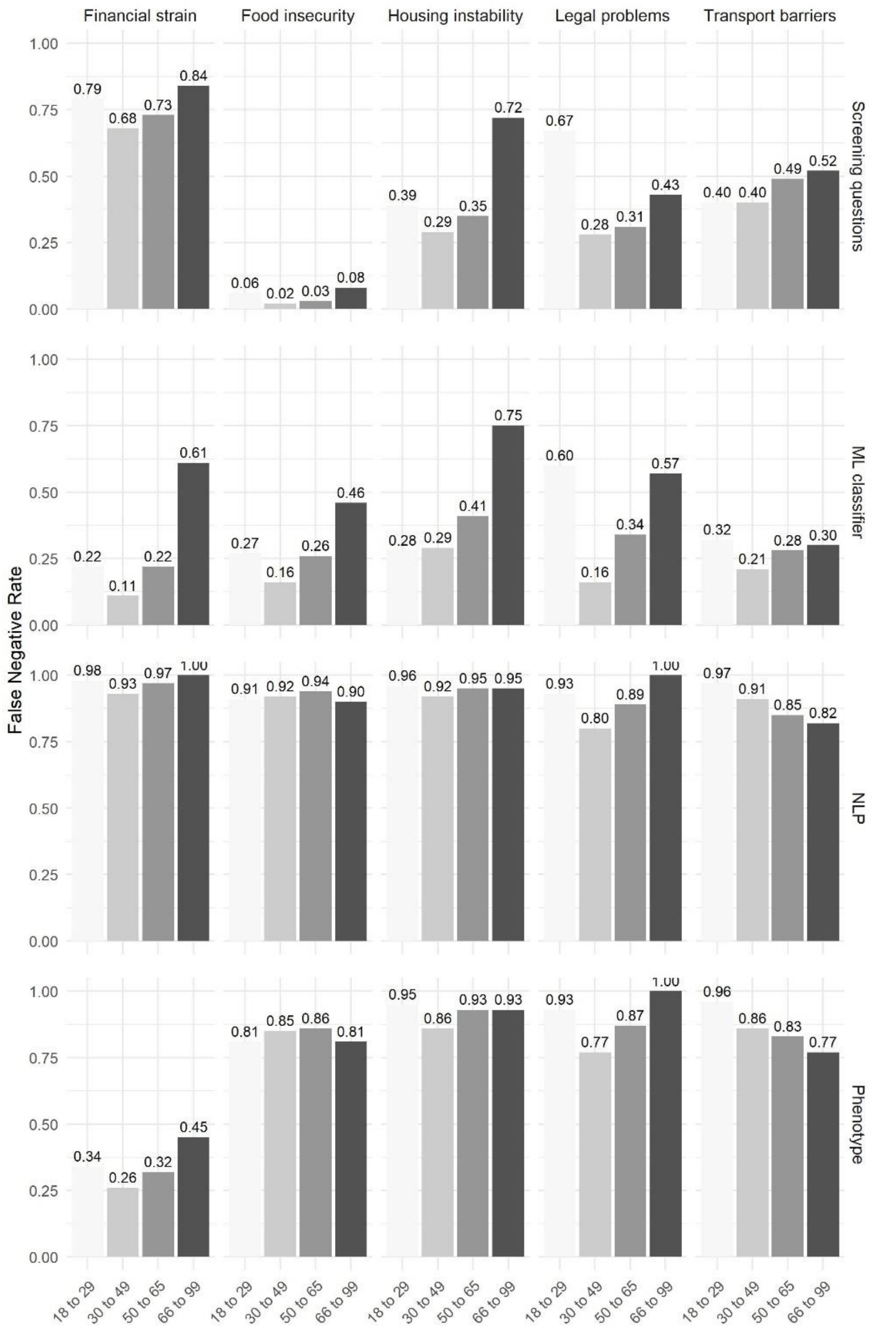

© 2025 Vest JR et al. *JAMA Network Open*.

eTable 1. List of reference standard outcomes, screening variables, and features used in ML classification and rule-based computable phenotype construction.

Feature	Description	% missing
Reference standards		
gold_housinginstability	Housing Instability Index	0.9
gold_foodinsecure	USDA. U.S. Household Food Security Survey Module	0.0
gold_financestrain	Consumer Financial Protection Bureau. CFPB Financial Well-Being Scale	1.8
gold_transport	Transportation barriers scale	0.0
gold_legal	Legal Status section of the Addiction Severity Index	0.9
EHR Screener		
epic_housinginstability	Epic: housing instability positive	1.5
epic_foodinsecure	Epic: food insecurity positive	1.1
epic_financestrain	Epic: financial strain positive	3.2
epic_transport	Epic: transportation barrier	0.8
epic_legal	Epic: legal problems social worker assessment	3.0
Used in Modeling & Computable Phenotypes		
lthighschool	Education less than high school equivalent	0.0
childrenhousehold	children <=18 present in household	0.0
language_ne_english	Preferred language not English	0.0
alias_present	Alias present in EHR	0.0
interpreter	Translations services used / Interpreter needed	0.0
phr	active EHR portal account	0.0
no_ssn	No social security number on file	0.0
no_email	No email on file	0.0

© 2025 Vest JR et al. *JAMA Network Open*.

no_emergency	no emergency contact on file	0.0
marital_married	marital status: married / partner / significant other	0.0
marital_single	marital status: single	0.0
marital_sepdev	marital status: separated or divorced	0.0
occup_unemp	occupation marked as unemployed	0.0
icdz_housing	Prior Housing insecurity ICD10 Z codes	0.0
icdz_financial	Prior Financial strain ICD10 Z codes	0.0
icdz_transport	Prior Transportation barriers ICD10 Z codes	0.0
icdz_food	Prior Food insecurity ICD10 Z codes	0.0
icdz_unemploy	Prior Unemployment ICD10 Z codes	0.0
icdz_legal	Prior Legal problems ICD10 Z codes	0.0
icd_legal	Prior Legal problems ICD10 codes (prison place of event & legal intervention)	0.0
icdz_any	Prior Any ICD10Z code present	0.0
icd_crime	Prior ICD codes for injuries related	0.0
icd_lead	Prior ICD10 code for lead poisoning	0.0
icdz_homeless	Prior Housing insecurity ICD10 Z codes limited to homelessness	0.0
icd_food	ICD10 food codes: failure to thrive, abnormal weight loss	0.0
overall_elixhauser	Prior Elixhauser comorbidity index score	0.0
screen_housing	Prior EHR screener identified housing instability	0.0
screen_financial	Prior EHR screener identified, financial strain	0.0
screen_food	Prior EHR screener identified food insecurity	0.0
screen_transport	Prior EHR screener identified transportation barriers	0.0
sw_legal	Prior Social worker assessment identified incarceration history	0.0
sw_unemploy	Prior Social worker assessment identified unemployment	0.0
sw_transport	Prior Social worker assessment identified transportation barriers	0.0
ref_social_worker	Prior Referral to social work	0.0
ref_financial	Prior Referral to financial counseling	0.0

© 2025 Vest JR et al. *JAMA Network Open*.

ref_food	Prior Referrals / Orders to WIC, Meals on Wheels, Cafeteria	0.0
ref_housing	Prior Patient directed to resources to address housing instability	0.0
ref_transportation	Prior Patient directed to resources to address transportation barriers	0.0
address_homeless	Address is known alias for homeless	0.0
address_shelter	Addresses linked to known shelter addresses	0.0
address_legal	Address is listed as prison / law enforcement	0.0
phone_homeless	Phone number a known homeless shelter	0.0
phone_legal	Phone number a known correction / jail	0.0
appointment_missed	number of missed appointments	0.0
appointment_kept	number of kept appointments	0.0
enc_fincoun	Prior visit with financial counseling (regardless	0.0
enc_sw	Prior visit with social worker (regardless	0.0
arrivaltaxi	mode of arrival Taxi or Public transportation	0.0
enc_totip	Total number of Inpatient admissions	0.0
enc_toted	Total number of ED visits	0.0
enc_totpc	Total number of primary care visits	0.0
enc_totbh	Total number of behavioral health visits	0.0
enc_totspec	total number of specialist visits	0.0
arrivdisch_legal	Mode of arrival was taxi or public transport	0.0
insurance_legal	Payer was justice system / law enforcement / corrections	0.0
insurance_uninsured	Uninsured or self-pay status	0.0
insurance_commercial	Payer was commercial / private	0.0
insurance_medicare	Payer was Medicare	0.0
insurance_2public	Change in insurance type from commercial to public/ Medicaid / dual / self-pay	0.0
insurance_2dual	Change in insurance type from Medicare to dual status	6.5

© 2025 Vest JR et al. *JAMA Network Open*.

underweight	BMI <18.5	0.0
phq9_dep	Prior Positive depression screen	0.0
phq9_anx	Prior Positive anxiety screen	0.0
tobacco	Tobacco usage (current)	0.0
ADI_STATERNK	area diversity index (10 worse)	0.0
hh_med_income	tract median household income (2020)	0.0
area_moved2lower	moved from a higher median income census tract to lower income during period	0.0
nlp_housing	NLP: housing instability	0.0
nlp_financial	NLP: finanaical strain	0.0
nlp_unemploy	NLP: unemployment	0.0
nlp_legal	NLP: legal problems	0.0
nlp_food	NLP: food insecurity	0.0
nlp_transport	NLP: transportation barriers	0.0
no_phone	No phone number	0.0
phone_change	Phone number changed in past 12 months	0.0
insurance_medicaid	Payer was Medicaid or other public	0.6
dual	Was dual (Medicare Medicaid) during study	0.0
address_change_str3	3 or more different addresses (street address)	0.0
enc_noshow	% of outpatient visits that were no shows	0.0
Used in bias assessments & descriptives		
age1829	age 18 to 29	0.0
age3049	age 30 to 49	0.0
age5065	age 50 to 65	0.0
age6699	age 66 and older	0.0
black_nh	Black non-Hispanic	0.0
white_nh	White non-Hispanic	0.0

© 2025 Vest JR et al. *JAMA Network Open*.

hispanic	Hispanic	0.0
othunk_nh	Other unknown	0.0
female	Female	0.0

From the EHR data, we created binary indicators for all elements that were direct measures of HRSNs (e.g., ICD-10 Z codes) or presence of a positive screen for a HRSN on a prior screening questionnaire. Following expert suggestions, we also created binary indicators of services that address HRSNs (e.g., referrals to social workers, orders for Medicaid taxis, etc.), or that were generally reflective of patients' social and economic position (e.g., changes to insurance status, no phone number). Missing values were classified as zero. We created binary indicators for median household income and Area Deprivation Index as highest quartile versus all others and replaced missing values with sample medians. We categorized age, created a binary indicator for female gender (compared to all others), and created a single race and ethnicity categorical variable following Office of Management & Budget guidelines.

© 2025 Vest JR et al. *JAMA Network Open*.

eTable 2. Prior performance of natural language processing algorithms.

The NLP algorithms to extract HRSNs were developed with the highest scientific rigor and published in the peer-reviewed literature[1,2]. A team of health informaticians, health services researchers, and data scientists from the Indiana University and the Regenstrief Institute developed FORCE (Finding Other Risks & Contexts Electronically) through a multiple stage process. First, the team established definitions of each of the target HRSNs based on the scientific literature. Second, the team assembled a training set of clinical documents from one multi-hospital health system in Indiana. This document corpus included multiple note types such as history & physical, progress notes, discharge summaries, operative reports, and others over a period of 6 months for adult patients. Initial keywords were identified through a review of n-grams. The team augmented the n-grams with additional keywords identified through a review of prior qualitative research on HRSNs, diagnostic code descriptions, and HRSN screening survey text. The training data were used for all keyword and rule-definitions. To test the performance of the algorithms, the team constructed an independent set of clinical documents from a second health system in the state. This step ensured the NLP algorithms were generalizable across systems and avoided any bias from development on a single data source. Manual review was used to determine performance. The Indiana University team re-deployed these algorithms in a third health system in Florida. Manual review was used to establish performance in the new health system. The NLP algorithms showed excellent performance on the validation dataset[1] suggesting generalizability of the product across health systems, geography, and patient populations. The performance of FORCE in developmental work is summarized, below[1,2].

Table B1. NLP performance in developmental datasets

HRSN	Sensitivity	Specificity	AUC
Financial strain	0.98	0.97	0.98
Food insecurity	0.98	0.93	0.96
Housing instability	1.00	0.95	0.98
Legal problems	0.99	0.93	0.96
Transportation barriers	0.99	0.84	0.92

1. Magoc T, Allen KS, McDonnell C, Russo JP, Cummins J, Vest JR, Harle CA. Generalizability and portability of natural language processing system to extract individual social risk factors. Int J Med Inf. 2023 Sep 1;177:105115. PMID: 37302362

2. Allen KS, Hood DR, Cummins J, Kasturi S, Mendonca EA, Vest JR. Natural language processing-driven state machines to extract social factors from unstructured clinical documentation. JAMIA Open. 2023 Jul 1;6(2):ooad024. PMCID: PMC10112959

© 2025 Vest JR et al. *JAMA Network Open*.

eTable 3. Rule-based health-related social needs computable phenotype definitions[1]

Construct definition	Data field	Values	Exclusions	Data processing steps / sources
**Housing instability** - housing disruptions or related problems, from frequent moves or difficulty paying rent to being evicted or being homeless	Patient address	Address listed as:<br>Homeless[1,2]<br>Shelter<br>None<br>Unknown<br>Don't know<br>No permanent<br>General delivery		
	Patient address	Address matches to a known:[2]<br>Shelter<br>Place of worship<br>Hospitals/EDs		Statewide lists identified through Dun & Bradstreet<br>US 8-Digit SIC Code:<br>83619901 - Destitute home<br>83619900 - Residential care, nec<br>83619903 - Halfway group home, persons with social or personal problems<br>866101 - Churches, temples, and shrines (limited to Indianapolis area)<br>8062 General Medical and Surgical Hospitals<br>NAICS 2022 Code:<br>62422 - Community Housing Services
	Diagnosis Codes (ICD10)	Z59.0-Z59.02 (homelessness)<br>Z59.1 (inadequate housing)<br>Z59.3<br>Z59.811-Z59.819 (housing)<br>Z59.89 (other economic and housing)<br>Z59.9 (unspecified economic and housing)		
	Prior SDOH / social history screening questions	Classification as high risk per screening algorithm (Epic)<br>1.In the last 12 months, was there a time when you were not able to pay the mortgage or rent on time?<br>2.In the last 12 months, how many places have you lived?<br>3.In the last 12 months, was there a time when you did not have a steady place to sleep or slept in a shelter (including now)?	Only positives. Negatives ignored: 1) focus on sensitivity and 2) factors change over time	
	Patient phone number	Number matches to known shelter		Statewide lists identified through Dun & Bradstreet<br>US 8-Digit SIC Code:<br>83619901 - Destitute home<br>83619900 - Residential care, nec

© 2025 Vest JR et al. *JAMA Network Open*.

				83619903 - Halfway group home, persons with social or personal problems<br>NAICS 2022 Code:<br>62422 - Community Housing Services
	Patient address	3 or more different addresses		Cleaning & translation steps:<br>1. Normalize addresses using https://github.com/GreenBuildingRegistry/usaddress-scourgify<br>2. Parse normalized addresses into standard components using https://github.com/datamade/usaddress<br>3. Geocode
	Clinical notes positive for housing instability	NLP algorithm[3,4]	Only positives. Negatives ignored: 1) focus on sensitivity and 2) factors change over time	
**Financial strain** –<br>the perceived ability of one's income to fulfil financial obligations; state of being wherein a person cannot fully meet current and ongoing financial obligations, cannot feel secure in their financial future, and is unable to make choices that allow them to enjoy life. Opposite of "well-being"	Insurance	Any means tested insurance coverage or no insurance (Medicaid, dual, other public insurance)		Local codes required.
	Referrals	Financial counseling		Local codes required.
	Diagnosis Codes (ICD10)	Z59.5 (extreme poverty)<br>Z59.6 (low income)<br>Z59.7 (insufficient social insurance & welfare support)<br>Z59.8 (other economic and housing)<br>Z59.9 (unspecified economic and housing)<br>Z91.110 (noncompliance due to financial hardship)<br>Z91.120 (noncompliance due to financial hardship)<br>Z91.190 (noncompliance due to financial hardship)<br>Z91.A10 (noncompliance due to financial hardship)<br>Z91.A20 (noncompliance due to financial hardship)<br>Z56.0 (unemployed)<br>Z59.0 -- .02 (homelessness)		
	Prior SDOH / social history screening questions	Classification as high risk per screening algorithm (Epic)<br>1. How hard is it for you to pay for the very basics like food, housing, medical care, and heating?	Only positives. Negatives ignored: 1) focus on sensitivity and 2) factors change over time	
	Referrals & orders	Means associated social services		Local codes required.

© 2025 Vest JR et al. *JAMA Network Open*.

	Homelessness indicators	Any housing instability indicator specific to homelessness	E.g. excludes Z59.1 (inadequate housing); Z59.2 (discord with neighbors); and SDOH / social screener	
	Clinical notes positive for financial strain	NLP algorithm[3,4]	Only positives. Negatives ignored: 1) focus on sensitivity and 2) factors change over time	
**Transportation** – including access to modes and transportation and burdens by distance / travel time	Diagnosis Codes (ICD10)	Z59.82 (transportation insecurity)<br><br>Z60.2 (living alone) AND<br>Z74.0 – Z74.9 (dependency /reduced mobility)		
	Referrals & orders	Order for transportation services		Local codes required.
	Prior SDOH / social history screening questions	Classification as high risk per screening algorithm (Epic)<br>1. Has the lack of transportation kept<br>you from medical appointments or from getting medications?<br>2. Has the lack of transportation kept you from meetings, work, or from getting things needed for daily living?	Only positives. Negatives ignored: 1) focus on sensitivity and 2) factors change over time	
	Mode of arrival	Arrival means values:<br>Taxi<br>Public transportation		Local codes required.
	Encounters	75% missed/cancelled/no show appointments		
	Clinical notes positive for transportation barriers	NLP algorithm[3,4]	Only positives. Negatives ignored: 1) focus on sensitivity and 2) factors change over time	
**Food Insecurity** – household lacks access to adequate food because of limited money or other resources	Referrals & orders	Food related services		Local codes required.
	Diagnosis Codes (ICD10)[5]	Z59.4 (Lack of adequate food)<br>Z59.41 (Food insecurity)<br>Z59.48 (Other specified lack of adequate food)<br>E63.9 Nutritional deficiency, unspecified		

© 2025 Vest JR et al. *JAMA Network Open*.

	Prior SDOH / social history screening questions	Classification as high risk per screening algorithm (Epic)<br>1. Within the past 12 months, have you worried that your food would run out before you got money to buy more?<br>2. Within the past 12 months, the food you bought just didn't last and you didn't have money to get more?	Only positives. Negatives ignored: 1) focus on sensitivity and 2) factors change over time	
	Diagnosis Codes (ICD10)	Financial strain positive AND<br>T73.0 (Starvation) or<br>X58.XXXA (external cause hunger) or<br>R62.51 (failure to thrive) or<br>R62.7 (failure to thrive) or<br>R63.4 (abnormal weight loss)		
	Clinical notes positive for food insecurity	NLP algorithm[3,4]	Only positives. Negatives ignored: 1) focus on sensitivity and 2) factors change over time	
**Legal problems** – criminal justice matters or events involving arrest and appearance in court to answer a charge	Diagnosis Codes (ICD10)	Z65.0 (conviction without imprisonment)<br>Z65.1 (imprisonment)<br>Z65.2 (release from prison)<br>Z65.3 (other legal)<br>Z02.89 (examination for prison)		
	Social worker assessment	Yes to either: Have you been convicted of a felony? or<br>Do you have any current legal issues?<br>(Epic EHR Social work screener)	Only positives. Negatives ignored: 1) focus on sensitivity and 2) factors change over time	
	Insurance	Payor is listed as Law enforcement		Local codes required.
	Diagnosis Codes (ICD10)	Y92.14 (prison place of injury)<br>Y35 (legal intervention)		
	Patient address	Is known associated with law enforcement<br>• Matching address<br>• Address includes<br>○ Jail<br>○ Prison		Local values / Dictionary<br>Local lists identified through Dun & Bradstreet with US 8-Digit SIC Code:<br>9223 - Correctional institutions<br>NAICS 2022 Code:<br>922140 - Correctional Institutions<br>92215 - Parole Offices and Probation Offices
	Admit/Discharge destination	Any justice system associated service location<br>• Court		Local codes required.

© 2025 Vest JR et al. *JAMA Network Open*.

		• Law enforcement		
	Clinical notes positive for legal problems	NLP algorithm[3,4]	Only positives. Negatives ignored: 1) focus on sensitivity and 2) factors change over time	

[1]All timeframes are within the past 12 months (and same day as survey)

References

1. Zech J, Husk G, Moore T, Kuperman GJ, Shapiro JS. Identifying homelessness using health information exchange data. J Am Med Inform Assoc. 2015 Mar 1;22(3):682–687.

2. Vickery KD, Shippee ND, Bodurtha P, Guzman-Corrales LM, Reamer E, Soderlund D, Abel S, Robertshaw D, Gelberg L. Identifying Homeless Medicaid Enrollees Using Enrollment Addresses. Health Serv Res. 2018 Jun;53(3):1992–2004. PMCID: PMC5980223

3. Allen KS, Hood DR, Cummins J, Kasturi S, Mendonca EA, Vest JR. Natural language processing-driven state machines to extract social factors from unstructured clinical documentation. JAMIA Open. 2023 Jul 1;6(2):ooad024. PMCID: PMC10112959

4. Magoc T, Allen KS, McDonnell C, Russo JP, Cummins J, Vest JR, Harle CA. Generalizability and portability of natural language processing system to extract individual social risk factors. Int J Med Inf. 2023 Sep 1;177:105115. PMID: 37302362

5. Arons A, DeSilvey S, Fichtenberg C, Gottlieb L. Documenting social determinants of health-related clinical activities using standardized medical vocabularies. JAMIA Open. 2019 Apr 1;2(1):81–88. PMCID: PMC6951949

eTable 4. Differential performance of measurement approaches for health-related social needs by demographic groups

**Measurement approach**	**Indication of differential performance**					
	**Gender**		**Race & Ethnicity**		**Age category**	
	**FN rate**	**AUC**	**FN rate**	**AUC**	**FN rate**	**AUC**
Screening questionnaire						
Food insecurity			■	□	■	
Housing instability	■	■			■	■
Financial strain					■	
Transportation barriers	□	□			◧	
Legal problems			■		■	
NLP						
Food insecurity			■			
Housing instability		□	◧	◧		
Financial strain			□		■	◧

© 2025 Vest JR et al. *JAMA Network Open*.

Transportation barriers					■	◧
Legal problems			◧	■	■	■
Rule-based computable phenotype						
Food insecurity			■			
Housing instability			◧	□	■	
Financial strain					■	
Transportation barriers					■	■
Legal problems			■	■	■	■
ML classification						
Food insecurity	□			■	■	
Housing instability			◧		■	
Financial strain				□	■	
Transportation barriers					□	
Legal problems	◧		◧	□	■	◧

Abbreviations: FN, False negative; AUC, Area Under the Curve.

**P* < .05 □

***P* < .01 ◧

****P* < .001 ■

© 2025 Vest JR et al. *JAMA Network Open*.
