# Supplementary material for: Performance of 4 Methods to Assess Health-Related Social Needs
Source: JAMA Netw Open. 2025 Aug 18;8(8):e2527426. doi: 10.1001/jamanetworkopen.2025.27426 (PMC12362220; doi:10.1001/jamanetworkopen.2025.27426)
Supplement: Supplement 2. — Data Sharing Statement [file jamanetwopen-e2527426-s002.pdf]

Data Sharing Statement

Vest. Performance of 4 Methods to Assess Health-Related Social Needs. *JAMA Netw Open*. Published August 18, 2025. doi:10.1001/jamanetworkopen.2025.27426

Data
Data available: No

Additional Information
Explanation for why data not available: Due to the data use restrictions put in place by consortium agreements among the health system partners, patient-level data cannot be shared or disseminated outside this project.
